# Outcomes following percutaneous coronary revascularization among South Asian and Chinese Canadians

**DOI:** 10.1186/s12872-017-0535-0

**Published:** 2017-04-19

**Authors:** Martha H. Mackay, Robinder Singh, Robert H. Boone, Julie E. Park, Karin H. Humphries

**Affiliations:** 1 School of Nursing, University of British Columbia, and St. Paul’s Hospital, 1081 Burrard St, Vancouver, BC V6Z 1Y6 Canada; 2 Faculty of Medicine, University of Manitoba and St. Boniface Hospital, Winnipeg, Canada; 3 Division of Cardiology, University of British Columbia and St. Paul’s Hospital, Vancouver, Canada; 4 BC Centre for Improved Cardiovascular Health, Vancouver, Canada; 5 Division of Cardiology, University of British Columbia, Vancouver, Canada

**Keywords:** Coronary artery disease, Percutaneous coronary intervention, Ethnicity, Outcomes

## Abstract

**Background:**

Previous data suggest significant ethnic differences in outcomes following percutaneous coronary revascularization (PCI), though previous studies have focused on subgroups of PCI patients or used administrative data only. We sought to compare outcomes in a population-based cohort of men and women of South Asian (SA), Chinese and “Other” ethnicity.

**Methods:**

Using a population-based registry, we identified 41,792 patients who underwent first revascularization via PCI in British Columbia, Canada, between 2001 and 2010. We defined three ethnic groups (SA, 3904 [9.3%]; Chinese, 1345 [3.2%]; and all “Others” 36,543 [87.4%]). Differences in mortality, repeat revascularization (RRV) and target vessel revascularization (TVR), at 30 days and from 31 days to 2 years were examined.

**Results:**

Adjusted mortality from 31 days to 2 years was lower in Chinese patients than in “Others” (hazard ratio [HR] 0.72; 95% confidence interval [CI] 0.53-0.97), but not different between SAs and “Others”. SA patients had higher RRV at 30 days (adjusted odds ratio [OR] 1.30; 95% CI: 1.12-1.51) and from 31 days to 2 years (adjusted hazard ratio [HR] 1.17; 95% CI: 1.06-1.30) compared to “Others”. In contrast, Chinese patients had a lower rate of RRV from 31 days to 2 years (adjusted HR 0.79; 95% CI: 0.64-0.96) versus “Others”. SA patients also had higher rates of TVR at 30 days (adjusted OR 1.35; 95% CI: 1.10-1.66) and from 31 days to 2 years (adjusted HR 1.19; 95% CI: 1.06-1.34) compared to “Others”. Chinese patients had a lower rate of TVR from 31 days to 2 years (adjusted HR 0.76; 95% CI: 0.60-0.96).

**Conclusions:**

SA had higher RRV and TVR rates while Chinese Canadians had lower rates of long-term RRV, compared to those of “Other” ethnicity. Further research to elucidate the reasons for these differences could inform targeted strategies to improve outcomes.

## Background

In Canada, the largest visible minority is South Asian (25%), followed by Chinese [1]. South Asians (SAs) are younger at presentation, and have higher rates of diffuse coronary artery disease (CAD) compared to non-SAs [2–7]. Differences in outcomes have been noted and may be explained by their higher prevalence of type-2 diabetes and modifiable risk factors such as smoking and obesity, or smaller coronary diameter and novel risk factors for CAD [7–10]. Conversely, Chinese have lower rates of atherosclerosis compared to others [11–13], but the prevalence among Chinese is increasing, attributed to increased dyslipidemia and other environmental influences [14]. A review of over 10 million deaths from the United States found Asian Indian men to have the highest proportional mortality ratio, followed by Asian Indian women and Filipino men, respectively [15]. In Canada, SA patients have paradoxically lower mortality rates following myocardial infarction (MI), despite a higher overall disease burden [16], whereas Chinese had higher short-term mortality following MI in one large cohort study [17].

Revascularization (coronary artery bypass grafting [CABG] or PCI) remains the mainstay of treatment for most patients with symptomatic CAD. Studies have shown that Canadian, British and Indian SAs experience poorer outcomes than non-SAs following CABG [18–22], especially those with diabetes [20]. British researchers have shown higher rates of re-stenosis, target lesion revascularization and CABG post-PCI among SAs versus non-SAs, but no differences in mortality [23, 24]. Canadian studies examining outcomes following acute MI among SA, Chinese and Caucasian patients have had conflicting findings regarding short-term mortality and recurrent MI [17, 21, 25] although a recent study demonstrated longer survival amongst acute coronary syndrome patients who received revascularization [26]. We aimed to compare the outcomes among men and women of SA, Chinese and “Other” ethnicity, following PCI.

## Methods

### Study design

This retrospective observational cohort study used prospectively collected data from the Cardiac Services British Columbia (CSBC) Cardiac Registry [27], a database including demographic, clinical and procedural outcome details (excluding mortality) of all patients undergoing cardiac procedures in the Canadian province of British Columbia. This included patients with elective (stable coronary disease), urgent (acute coronary syndrome) and emergent (ST-elevation MI) urgency ratings. Mortality data was obtained from the Vital Statistics Agency of British Columbia [28]. We included all patients over 20 years who had undergone PCI in British Columbia as their first revascularization, from April 1, 2001 to October 31, 2010.

Patients with a prior PCI or CABG were excluded for accurate identification of those who required any repeat revascularization (RRV) or target vessel revascularization (TVR). The study received approval from the institution’s Research Ethics Board.

### Measures

Demographic data and procedural details were obtained from the CSBCCR. Ethnicity was assigned by CSBC using the Nam Pechan surname analysis program for SA ethnicity (Bradford Health Authority, Bradford, UK), (86–92% sensitivity; greater than 95% specificity) [29, 30] and Quan’s List for Chinese ethnicity [31] (78% sensitivity; 99.7% specificity; 81% positive predictive value; 99.6% negative predictive value). Patients whose names were not identified as either SA or Chinese were classified as “Other”; Canadian census data indicate that approximately 97% of Canadians who do not report SA or Chinese ethnicity are of European ancestry [1]. After surname analysis, the dataset was stripped of all patient identifiers.

Three endpoints (mortality, RRV and TVR) were determined at two time points (30 days and 31 days to 2 years). If a patient experienced more than one RRV, the first procedure was taken as the event. Staged PCI was not considered a RRV. We defined staged PCI as an elective procedure performed within 60 days after the index PCI on a different vessel than that of the index PCI. Furthermore, TVR was defined as a RRV on the same vessel as the index PCI. Four coronary arteries (left main, left anterior descending, left circumflex and right) were used to define staged PCI and TVR.

### Statistics

Group differences were assessed using Chi-squared tests for categorical variables and analysis of variance for continuous variables after log transformation, since neither age nor body mass index (BMI) were normally distributed. For 30-day event rates, proportions were calculated, whereas Kaplan-Meier estimates were used to calculate 31-day to 2-year event rates, due to different lengths of follow-up.

Since the hazard ratios (HR) for ethnicity in the early (first 30-days) compared to the late (31 days to 2 years) differed, two separate analyses were performed to further examine ethnicity-based differences in the primary outcomes: logistic regression analysis (up to 30 days) and Cox proportional hazard models (after 30 days), with “Others” as the reference group. When examining repeat revascularization and TVR, patients were censored at the time of death or at the end of two years, whichever came first.

The following clinical covariates were included in the adjusted models: age, BMI, smoking status (current, former, never), prior infarction, history of hypertension, dyslipidemia, cerebrovascular disease, congestive heart disease, diabetes mellitus, peripheral vascular disease, pulmonary disease, liver/gastrointestinal disease, malignancy, dialysis, left ventricular ejection fraction, use of ASA, ACE inhibitor or statin in 24 h before the procedure, as well as peri-procedural variables, including indication (acute coronary syndrome, stable angina vs. other), procedure urgency (elective vs. non-elective), disease severity (3-vessel or left main vs. rest). Due to high missing rates of stent type and cardiogenic shock, additional analysis was performed, including these variables in the adjusted model. Sex was included in the final model regardless of its significance. When the sex effect was significant, sex by ethnicity interaction was then added to see if the effect of ethnicity on outcomes was modified by sex. When a covariate violated the proportional hazard assumption, it was used as a stratifying variable or an interaction term with a time variable was added. We used SAS version 9.3 (SAS Institute Inc., Cary, NC) for all analyses.

## Results

### Clinical and demographic characteristics

41,792 patients who underwent PCI as a first revascularization were included, of which 3904 (9.3%) were of SA, 1345 (3.2%) Chinese, and 36,543 (87.4%) “Other” ethnicity. There were many statistically significant differences among the three groups (see Table 1), though not all are clinically significant. In terms of the urgency of the PCI, Chinese patients were most likely to undergo an elective procedure, whereas patients of “Other” ethnicity were least likely.

**Table 1:** Demographic and pre-procedure clinical characteristics

Variable^a^	All	Ethnicity			*p-value*
		SA (*n* = 3904)	Chinese (*n* = 1345)	Other (*n* = 36,543)	
Male	30047 (71.9)	2741 (70.2)	1018 (75.7)	26288 (71.9)	<0.001
Age	64 (56, 73)	62 (53.5, 71)	65 (56, 74)	64 (56, 74)	<0.001
BMI, kg/m^2^	27.3 (24.6, 30.5)	26.2 (23.9, 29.1)	24.5 (22.4, 26.7)	27.5 (24.9, 30.8)	<0.001
Elective	9534 (22.9)	900 (23.1)	362 (27.1)	8272 (22.7)	<0.001
Indication					
ACS Stable angina Other	30115 (72.3) 9877 (23.7) 1662 (4.0)	2780 (71.4) 976 (25.1) 140 (3.6)	866 (64.5) 416 (31.0) 60 (4.5)	26469 (72.7) 8485 (23.3) 1462 (4.0)	<0.001
Medications within 24 h prior to procedure					
ASA ACE-I Statins	38030 (91.0) 20294 (48.6) 24910 (59.6)	3543 (90.8) 1735 (44.4) 2306 (59.1)	1156 (85.9) 524 (39) 751 (55.8)	33331 (91.2) 18035 (49.4) 21853 (59.8)	<0.001 <0.001 0.011
Congestive heart failure	2320 (5.6)	232 (5.9)	84 (6.3)	2004 (5.5)	0.260
Prior MI	7094 (17)	580 (14.9)	169 (12.6)	6345 (17.4)	<0.001
HTN	23744 (56.8)	2310 (59.2)	892 (66.3)	20542 (56.2)	<0.001
Dyslipidemia	22346 (53.5)	2281 (58.4)	751 (55.8)	19314 (52.9)	<0.001
CBVD	2762 (6.6)	203 (5.2)	85 (6.3)	2474 (6.8)	0.01
Diabetes Mellitus	8817 (21.1)	1204 (30.8)	353 (26.2)	7260 (19.9)	<0.001
Dialysis	520 (1.2)	56 (1.4)	28 (2.1)	43 (1.2)	0.008
LVEF					
> 50% 30–50% < 30% Not entered	20570 (49.2) 15893 (38.0) 1065 (2.6) 4264 (10.2)	2128 (54.5) 1285 (32.9) 129 (3.3) 362 (9.3)	715 (53.2) 429 (31.9) 36 (2.6) 165 (12.3)	17727 (48.5) 14179 (38.8) 900 (2.5) 3737 (10.2)	<0.001
Smoking status					
Current	9149 (21.9)	445 (11.4)	185 (13.8)	8519 (23.3)	<0.001
Former	15926 (38.1)	546 (14)	357 (26.5)	15023 (41.1)	
Never	16717 (40)	2913 (74.6)	803 (9.7)	13001 (35.6)	
PVD	2847 (6.8)	120 (3.1)	46 (3.4)	2681 (7.3)	<0.001
Pulmonary disease	4190 (10)	304 (7.8)	82 (6.1)	3804 (10.4)	<.0001
Liver-GI	3492 (8.4)	305 (7.8)	105 (7.8)	3082 (8.4)	0.3126
Malignancy	2898 (6.9)	126 (3.2)	63 (4.7)	2709 (7.4)	<.0001
3-vessel or left main disease	9445 (22.7)	1009 (26.1)	356 (26.7)	8080 (22.4)	<0.001

*SA* South Asian, *BMI* body mass index, *ACS* acute coronary syndrome, *MI* myocardial infarction, *HTN* hypertension, *CBVD* cerebrovascular disease, *LVEF* *left* ventricular ejection fraction, *PVD* peripheral vascular disease, *GI* gastrointestinal

^a^For age and BMI, median and interquartile ranges are reported; for other variables frequency and percentages are reported

### Mortality

Patients of Chinese ethnicity had higher crude rates of 30-day mortality (*n* = 48, rate = 3.6%; 95% CI: 2.6-4.6) compared to both the SA group (*n* = 83, rate = 2.1%; 95% CI: 1.7-2.6) and “Others” (*n* = 799, 2.2%; 95% CI: 2.0-2.3). There was no difference after adjustment in 30-day mortality between Chinese and “Others” (OR 1.18; 95% CI: 0.84-1.66), or SAs and “Others” (OR 0.88; 95% CI: 0.68-1.13) (see Table 2). There were no sex differences in 30-day mortality (OR 1.02; 95% CI 0.88-1.20). Unadjusted 31-day to 2-year mortality was lower in SA Canadians as compared to Chinese patients or Others; however, in adjusted models there was no statistically significant difference in 31-day to 2-year mortality between SA patients and “Others” (HR 0.96; 95% CI: 0.79-1.16), but Chinese patients had lower mortality compared with “Others” (HR 0.72; 95% CI: 0.53-0.97). There were no significant sex differences found in mortality during this period, after adjustment (HR 1.02; 95% CI: 0.91-1.13).

**Table 2:** Unadjusted and adjusted odd ratios and hazard ratios of mortality, by ethnicity and sex

Characteristic		Mortality			
		30-Day^a^		31 Days to 2 Years	
		Unadjusted OR (95% CI)	Adjusted^d^ OR (95% CI)	Unadjusted HR (95% CI)	Adjusted^d^ HR (95% CI)
Ethnicity^b^	SA *n* = 3904	0.96 (0.75,1.22)	0.88 (0.68, 1.13)	0.76 (0.63, 0.92)	0.96 (0.79, 1.16)
	Chinese *n* = 1345	1.64 (1.20, 2.23)	1.18 (0.84, 1.66)	0.85 (0.63, 1.14)	0.72 (0.53, 0.97)
Sex^c^		1.48 (1.29, 1.71)	1.02 (0.88,1.20)	1.44 (1.31, 1.60)	1.02 (0.91, 1.13)

*OR* odds ratio, *CI* confidence interval, *SA* South Asian

^a^For 30-day repeat revascularization, odds ratio (OR) and its CI are reported; for 31-day to 2-year outcomes, hazard ratio (HR) and its CI are reported

^b^ Referent : “Others” (*n* = 36,543)

^c^ Referent: Male

^d^Adjusted for urgency (elective vs. non-elective), indication (acute coronary syndrome, stable angina vs. other), ASA, ACE-inhibitor, statin use within 24 h prior to procedure, smoking status (current, former vs. never), prior infarction, hypertension, dyslipidemia, cerebrovascular disease, diabetes mellitus, peripheral vascular disease, pulmonary disease, liver-gastrointestinal disease, malignancy number of diseased vessel (3 or left main vs. rest), dialysis, left ventricular ejection fraction (>50% 30–50%, <30%, vs. not entered), age and BMI

### Repeat revascularization

Patients of SA ethnicity had higher crude rates of 30-day repeat revascularization (*n* = 258, rate = 6.6%; 95% CI: 5.8-7.4) compared to both the Chinese group (*n* = 58, rate = 4.3%; 95% CI: 3.2-5.4) and “Others” (*n* = 1698, rate = 4.6%; 95% CI: 4.4-4.9). With adjustment, SA patients rate of RRV remained 30% higher at 30 days (OR 1.30; 95% CI: 1.12-1.51), compared to “Others” (see Table 3). There was no difference in rates of 30-day RRV between Chinese and “Others”.

**Table 3:** Unadjusted and adjusted odd ratios and hazard ratios of repeat revascularization, by ethnicity and sex

Characteristic		Repeat Revascularization			
		30-Day^a^		31 Days to 2 Years	
		Unadjusted OR (95% CI)	Adjusted^d^ OR (95% CI)	Unadjusted HR (95% CI)	Adjusted^d^ HR (95% CI)
Ethnicity^b^	SA *n* = 3904	1.44 (1.25, 1.66)	1.30 (1.12, 1.51)	1.24 (1.13, 1.37)	1.17 (1.06, 1.30)
	Chinese *n* = 1345	0.98 (0.75, 1.27)	0.88 (0.67, 1.16)	0.83 (0.68, 1.00)	0.79 (0.64, 0.96)
Sex^c^		1.44 (1.25, 1.65)	0.85 (0.76, 0.95)	0.96 (0.90, 1.03)	0.99 (0.92, 1.07)

*OR* odds ratio, *CI* confidence interval, *SA* South Asian

^a^For 30-day repeat revascularization, odds ratio (OR) and CI are reported. For post 30-day 2-year outcomes, hazard ratio (HR) and CI are reported

^b^ Referent: “Others” (*n* = 36,543)

^c^ Referent: Male

^d^ Adjusted for urgency (elective vs. non-elective), indication (acute coronary syndrome, stable angina vs. other), ASA, ACE-inhibitor, statin use within 24 h prior to procedure, smoking status (current, former vs. never), prior infarction, hypertension, dyslipidemia, cerebrovascular disease, diabetes mellitus, peripheral vascular disease, pulmonary disease, liver-gastrointestinal disease, malignancy number of diseased vessel (3 or left main vs. rest), dialysis, left ventricular ejection fraction (>50% 30–50%, <30%, vs. not entered), age and BMI

Although women had lower rates of 30-day RRV compared to men on multivariate analysis (OR 0.85; 95% CI 0.76-0.95), there was no significant sex-by-ethnicity interaction on RRV (*p* interaction = 0.91).

SA patients also had significantly higher crude rates of RRV from 31 days to 2 years, as compared to “Others” or Chinese patients. On multivariate analysis, SA patients’ RRV remained significantly higher (HR 1.17; 95% CI: 1.06-1.30); in contrast, Chinese patients had a 21% lower adjusted rate of RRV (HR 0.79; 95% CI: 0.64-0.96). There was no sex difference in RRV from 31 days to 2 years post-procedure.

### Target vessel revascularization

SA patients (*n* = 127, rate = 3.3%; 95% CI: 2.8-3.9) had significantly higher rates of TVR at 30 days compared to both the Chinese (*n* = 27, rate = 2.1%; 95% CI: 1.3-2.8) and Other patients (*n* = 836, rate = 2.3%; 95% CI: 2.2-2.5). After adjustment, SA patients had a 35% higher rate of 30-day TVR compared to “Others” (OR 1.35; 95% CI: 1.10-1.66), but no difference in 30-day TVR between Chinese patients and “Others” (see Table 4). There was no sex difference in TVR for the first 30 days.

**Table 4:** Unadjusted and adjusted odd ratios and hazard ratios of target vessel revascularization, by ethnicity and sex

Characteristic		Target Vessel Revascularization			
		30-Day^a^		31 Days to 2 Years	
		Unadjusted OR (95% CI)	Adjusted^d^ OR (95% CI)	Unadjusted HR (95% CI)	Adjusted^d^ HR (95% CI)
Ethnicity^b^	SA *n* = 3904	1.46 (1.20, 1.77)	1.36 (1.10, 1.66)	1.25 (1.12, 1.40)	1.10 (1.06, 1.34)
	Chinese *n* = 1345	0.92 (0.63, 1.36)	0.83 (0.56, 1.23)	0.80 (0.64, 1.01)	0.76 (0.60, 0.96)
Sex^c^		0.81 (0.70, 0.94)	0.86 (0.73, 1.00)	1.00 (0.93, 1.09)	1.03 (0.95, 1.12)

^a^For 30-day repeat revascularization, odds ratio (OR) and confidence intervals (CI) are reported; for post-30-day 2-year outcomes, hazard ratio (HR) and CI are reported

^b^ Referent: “Others” (*n* = 36,543)

^c^ Referent: Male

^d^ Adjusted for urgency (elective vs. non-elective), indication (acute coronary syndrome, stable angina vs. other), ASA, ACE-inhibitor, statin use within 24 h prior to procedure, smoking status (current, former vs. never), prior infarction, hypertension, dyslipidemia, cerebrovascular disease, diabetes mellitus, peripheral vascular disease, pulmonary disease, liver-gastrointestinal disease, malignancy number of diseased vessel (3 or left main vs. rest), dialysis, left ventricular ejection fraction (>50% 30–50%, <30%, vs. not entered), age and BMI

SA patients also had higher crude rates of TVR between 31 days to 2 years than Chinese patients or “Others”. Following adjustment, SA patients also had a significantly higher rate of TVR from 31 days to 2 years (HR 1.19; 95% CI: 1.06-1.34). However, Chinese patients were found to have a lower adjusted rate of TVR, compared to “Others” (HR 0.76; 95% CI: 0.60-0.96). There were no sex differences in TVR between 31 days and 2 years.

## Discussion

To our knowledge, this is the largest Canadian cohort study investigating the association of ethnicity with outcomes of *all* patients who have undergone PCI (i.e., of elective, urgent and emergent urgency). Due to the large sample size, we found many statistically significant differences in baseline characteristics. Many of these differences were small and their clinical relevance is unclear. The younger age of SAs at presentation for initial PCI and the higher rate of diabetes are consistent with previous reports [11, 14, 16–19, 22–24]. Interestingly, the “Other” ethnicity cohort had the lowest rates of diabetes, hypertension and dyslipidemia, yet had the highest rates of vascular disease. This might be explained by their having the highest rates of current and former smoking.

Finding no sex differences in any of the specified PCI outcomes in our overall sample is consistent with some other researchers’ findings [23, 24, 32]. However, others have shown that, among patients undergoing angiogram for either stable angina or ACS, women have worse outcomes, even after adjusting for revascularization [33, 34]. Identifying other factors (e.g. microvascular dysfunction, diffuse distal disease, higher rate of depression) that might explain these conflicting findings demands further study.

There were higher rates of RRV and TVR between 30 days and 2-years among SA patients, and lower among Chinese. British and other investigators have similarly found significantly higher rates of TVR in SA patients [18–20, 22–24]. Given our adjustment for traditional risk factors and relevant covariates, novel risk factors and smaller coronary diameters may explain these differences [7–10]. However, Anand et al. have shown SA Canadians have higher atherosclerosis and cardiovascular event rates, compared to those of European and Chinese background [14], despite adjusting for traditional risk factors, Framingham risk and novel factors (fibrinogen, plasminogen activator inhibitor- 1, lipoprotein (a), homocysteine). These findings, taken with ours, suggest the possibility of an unknown pathophysiological mechanism of accelerated atherosclerotic disease in patients of SA ethnicity. Our population-based findings indicate no difference in post-PCI mortality among the three groups, which is similar to other reports [32]. We found a non-significant trend toward higher short-term mortality in Chinese patients, which others have found [17, 21]. King et al. reported a tendency among Chinese patients toward atypical symptoms and delayed treatment-seeking for symptoms of ACS, which may explain the trend toward higher short-term mortality [35]. Other explanations may include higher procedural complications or bleeding rates [36]. Chinese patients in our study had significantly lower rates of RRV and TVR from 30 days to 2 years. A prior study examined variations in outcomes among Chinese, Indian Asian and Malay patients following PCI [12] and found Chinese patients had significantly lower rates of MI, RRV and mortality compared to SA patients. Previous Canadian studies have found similarly lower rates of MI and recurrent events among Chinese MI patients, but higher short-term mortality [17, 37]. We were unable to find any literature comparing post-PCI outcomes of Chinese patients to a cohort of patients of European ancestry.

Our study has limitations. Although we used validated surname analysis tools to identify ethnicity [29–31], self-report remains the gold standard [38, 39]. Misclassification of ethnicity could lead to a bias towards the null. Some peri-procedural factors were not available for inclusion in the model including ST-elevation MI as the specific indication for PCI and ejection fraction. We also did not have data on socioeconomic status, or post-PCI medical therapy and risk factor control, any of which might have had an effect on outcomes.

## Conclusions

Although mortality rates following PCI are similar among ethnic groups, SAs have higher rates of RRV and TVR as compared to those of “Other” ethnicity. Conversely, Chinese patients had lower rates of RRV and TVR compared to those of “Other” ethnicity. Further investigation of ethnicity-based variability in PCI outcomes is warranted.

## Abbreviations

BMI: body mass index
CABG: coronary artery bypass graft
CAD: coronary artery disease
CI: confidence interval
HR: hazard ratio
MI: myocardial infarction
OR: odds ratio
PCI: percutaneous coronary intervention
RRV: repeat revascularization
SA: South Asian
TVR: target vessel revascularization
